# DREAM4: Combining Genetic and Dynamic Information to Identify Biological Networks and Dynamical Models

**DOI:** 10.1371/journal.pone.0013397

**Published:** 2010-10-25

**Authors:** Alex Greenfield, Aviv Madar, Harry Ostrer, Richard Bonneau

**Affiliations:** 1 Computational Biology Program, New York University Sackler School of Medicine, New York, New York, United States of America; 2 Department of Biology, Center for Genomics and Systems Biology, New York University, New York, New York, United States of America; 3 Human Genetics Program, Department of Pediatrics, New York University Langone Medical Center, New York, New York, United States of America; 4 Computer Science Department, Courant Institute of Mathematical Sciences, New York University, New York, New York, United States of America; Center for Genomic Regulation, Spain

## Abstract

**Background:**

Current technologies have lead to the availability of multiple genomic data types in sufficient quantity and quality to serve as a basis for automatic global network inference. Accordingly, there are currently a large variety of network inference methods that learn regulatory networks to varying degrees of detail. These methods have different strengths and weaknesses and thus can be complementary. However, combining different methods in a mutually reinforcing manner remains a challenge.

**Methodology:**

We investigate how three scalable methods can be combined into a useful network inference pipeline. The first is a novel t-test–based method that relies on a comprehensive steady-state knock-out dataset to rank regulatory interactions. The remaining two are previously published mutual information and ordinary differential equation based methods (tlCLR and Inferelator 1.0, respectively) that use both time-series and steady-state data to rank regulatory interactions; the latter has the added advantage of also inferring dynamic models of gene regulation which can be used to predict the system's response to new perturbations.

**Conclusion/Significance:**

Our t-test based method proved powerful at ranking regulatory interactions, tying for first out of 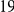 methods in the DREAM4 100-gene *in-silico* network inference challenge. We demonstrate complementarity between this method and the two methods that take advantage of time-series data by combining the three into a pipeline whose ability to rank regulatory interactions is markedly improved compared to either method alone. Moreover, the pipeline is able to accurately predict the response of the system to new conditions (in this case new double knock-out genetic perturbations). Our evaluation of the performance of multiple methods for network inference suggests avenues for future methods development and provides simple considerations for genomic experimental design. Our code is publicly available at http://err.bio.nyu.edu/inferelator/.

## Introduction

Predicting how a cell will respond, at the molecular level, to environmental and genetic perturbations is a key problem in systems biology. Molecular regulatory systems-level responses are governed by several regulatory mechanisms including the underlying transcriptional regulatory network (RN). Recently, there has been an increase in the number of genome-wide datasets appropriate for large scale network inference, which has driven a large interest in methods for learning regulatory networks from these datasets. In general, the question of inferring a transcriptional RN can be posed in the following way: given a set of regulators (transcription factors - TFs) and a set of targets (genes), what are the regulatory relationships between the elements in these two sets? These relationships can be directed (e.g. gene A regulates gene B) or undirected (e.g. there is a regulatory relationship between gene A and gene B), and can have parameters describing the strength, confidence and/or kinetics of the regulatory interaction (depending on the method used). RN inference techniques use three main types of genome-wide data: 1) steady-state transcriptional profiling of the response to perturbations (e.g. gene knock-out or exposure to a drug,), 2) collections of time series observations following relevant perturbations, and 3) measurements of TF-DNA binding. Different types of RN inference methods produce RNs that vary in detail and comprehension. One critical distinction is the scalability of any given method. Typically, methods that learn less detailed regulatory models scale to larger systems and data sizes than methods that learn more complex models. Another critical difference between methods is whether causal (directed) edges or undirected relationships are learned. Several current methods aim to learn dynamical parameters, such as TF-target activation rates and rates of degradation of gene products. Ideally, a computational biologist should choose the most detailed method that the data will support, as more detailed models can suggest more focused biological hypothesis and be used to model a system's behavior in ways that simple network models cannot. Given this constant need to balance the specific features of any given biological dataset with the capabilities of multiple RN inference algorithms, testing of RN inference methods using a variety of datasets is a critical field-wide activity. Several recent methods aim to do so by generating biologically meaningful datasets with a known underlying topology [1]–[4].

To this end, the Dialogue for Reverse Engineering Assessments and Methods (DREAM) [5] provides a set of networks which can be used to develop and test RN inference methods. The networks presented by DREAM make some simplifications of the networks found in a cell, and the corresponding datasets are ideal in their completeness. The control of cellular processes occurs on at least four distinct levels including DNA, transcript, protein, and metabolite. Measuring only transcript levels ignores the fact that cellular interactions happen on the level of proteins, and are mediated in many cases by metabolites. Accordingly, an ideal dataset for RN inference would contain time-series measurements of multiple levels of regulation (RNA, protein, protein-modifications, etc.) with the sampling rate on the order of the fastest reaction. Additionally, the cellular response to genetic perturbation (e.g. gene knock-out) would also be available. Although advances are currently being made in the cost and accuracy of genome-wide proteomics, metabolomics, and protein binding (ChIP-chip, ChIP-seq) [6], [7] measurements, the most mature and cost efficient technologies remain those that measure genome-wide transcription-level responses. Experimental and financial constraints typically prohibit obtaining these measurements in a finely time-resolved manner. The DREAM challenge removes many of these constraints and presents participants with an idealized expression dataset for which the true topology (gold-standard) is known. This presents a unique opportunity to develop RN inference methods and immediately test their performance by comparison with the gold-standard.

It should be noted that biological systems present several advantages not relevant to the DREAM4 challenge. These advantages (not discussed here) are leveraged by integrative methods for learning modularity prior to inference [8]–[12], methods that use structured priors derived from compilations of validated biological regulatory interactions [13]–[16], and approaches to characterize binding sites [17], [18]. A thorough review of current network inference methods is beyond the scope of this introduction but can be found in [19]–[24]. Here we briefly review only the classes of methods that we utilized in our hybrid approach: mutual information (MI) based methods, ordinary differential equation (ODE) based methods, and resampling methods.

Several methods for detecting significant regulatory associations are based on similarity metrics derived from information theory, such as MI. [25]. The MI between two signals (in this case the expression of a TF and its target) is calculated by subtracting the joint entropy of each signal from the sum of their entropies. It is similar to correlation (the higher the magnitude, the stronger the relationship), but is more generally applicable as it does not assume a linear relationship between the two signals, nor does it assume continuity. At their core, methods that rely on MI generally infer undirected interactions, as the MI between two variables is a symmetric quantity [26]–[29], however modifications can be made that allow for the inference of direction [30], [31]. Here, we use an MI-based method, time-lagged Context Likelihood of Relatedness (tlCLR) [31], which is based on Context Likelihood of Relatedness (CLR) [29], to learn initial topology that is further optimized and parametrized by Inferelator 1.0 [32]. tlCLR extends CLR by making use of the temporal information contained in time series observations to estimate the directionality of a significant regulatory interaction. This method is described in [31] and is reviewed in the methods section. tlCLR cannot be used to predict the response of the system to previously unseen perturbations as it does not infer any dynamical parameters. A different approach is needed to calculate these dynamical parameters. In the context of our full RN inference pipeline, which includes fitting of dynamical parameters, tlCLR is used as a feature selection algorithm that identifies a set of likely regulators for each target based on time-lagged, corrected MI.

Ordinary differential equation based methods for RN inference attempt to learn not only the topology of the network (i.e. “who regulates who”), but also the dynamical parameters associated with each regulatory interaction. Regulatory network models resulting from these methods can be used to predict the system-wide response to previously unseen conditions, future time-points, and the effects of removing system components. A drawback of these methods is that they generally require time-series data and more complete datasets than many alternative methods. ODE methods model the rate of change in the expression of a gene as a function of TFs (and other relevant effects) in the system. ODE based methods differ in their underlying functional forms, how the ODE system of equations is solved (coupled or uncoupled solution), and how prior knowledge and sparsity constraints are imposed on the overall inference procedure. For example, several methods have been proposed that use complex functional forms [33], and solve a coupled system [33], [34], while other methods [32], [35]–[38] solve a simplified linear system of ODEs. The Inferelator 1.0 [32], is an RN inference method which learns the network as a system of linear ODEs, where the rate of change for each gene is modeled as a function of the known regulators in the system. Inferelator 1.0 uses a finite difference approximation to estimate the change in the response over a given time interval, and uses an efficient implementation of 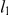-constrained linear regression, LARS [39], to enforce model sparsity. The Inferelator 1.0 has previously been used to learn a large portion of the *Halobacterium salinarium* transcriptional regulatory network, and was able to predict mRNA levels of 85% of the genes in the genome over new experimental conditions [40]. Additionally, feature selection by tlCLR followed by optimization and parameterization via Inferelator 1.0 was a top performing method for the DREAM3 network challenge [31]. One drawback of the original formulation of these scalable MI and ODE based methods is that they rely on point estimates for many network parameters and thus are not ideal for estimating the error in the inferred parameters [41]. One possible solution is to use a resampling approach [42], [43] to generate an ensemble of predicted networks from which the confidence interval for any parameter can be estimated.

Resampling refers to a broad class of statistical methods that are often used to assess confidence bounds on sample statistics by empirically generating distributions [42]. Recently, several groups have used resampling approaches in a biological context. In this setting resampling methods are an attractive means of determining confidence bounds on model parameters (such as the strength and directionality of a putative regulatory interaction) for two main reasons: 1) resampling methods are non parametric and thus applicable in cases where complex or ill-understood regulatory relationships might confound assumptions about the correct error distribution, and 2) resampling methods do not, in our case, decrease algorithm scalability. Resampling methods have been applied in several contexts to estimate error in a variety of genomics data-analysis contexts. Kerr et al. [44] used a resampling approach to assess confidence bounds of clusters from ANOVA models. Resampling of a gaussian process regression model was used by Kirk et al. [45] to show the sensitivity of the inferred network to uncertainty in the underlying data. Friedman et al. [46] used a resampling approach of a Bayesian network reconstruction algorithm to assess the confidence of inferred parameters. Additionally, Marbach et al. [47] showed that a resampling approach applied to a genetic algorithm for network inference was a top performering method in the DREAM2 five-gene network challenge. We show that by using a resampling approach to generate ensembles of networks with our network inference pipeline we can improve the accuracy of our topology predictions.

Here we focus on which data types (time-series or steady-state), and which methods (ODE-based, MI-based, genetic perturbation based, or combinations thereof) can be expected to perform best at either reconstructing network topology or predicting the response of the system to new perturbations. Our analysis suggests several simple considerations for determining the correct balance between time-series and steady-state data required for large-scale network inference, and how to use these distinct data types in a mutually reinforcing manner.

## Methods

**Figure 1:**
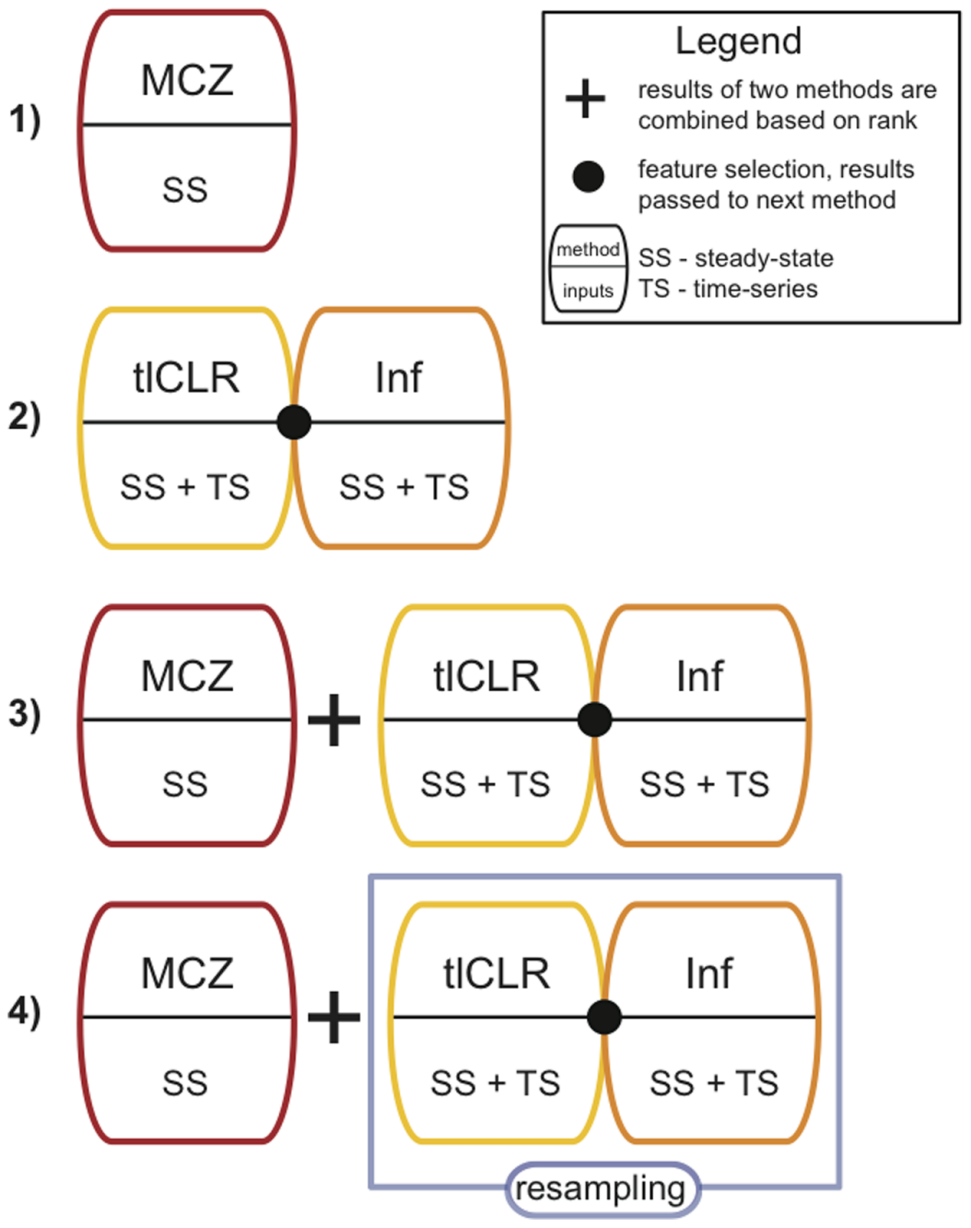
Network inference pipelines tested. We developed and tested four network inference pipelines composed of the methods described in Table 1. Pipeline 1, MCZ, uses median corrected z-scores on the steady state genetic knock-out data (eq. 2). We submitted topology predictions from this method, tying for 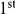 place. Pipeline 2, tlCLR-Inferelator, (eq. 19) uses tlCLR as a feature selection procedure, followed by further model selection and parameterization by the Inferelator 1.0. This pipeline was previously published and placed 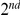 for the DREAM3 network inference competition [31]. Pipeline 3, tlCLR-Inferelator+MCZ, (eq. 20, developed to test the complementarity of the topology predictions made by pipelines 1 and 2). Pipeline 3 combines the results form pipelines 1 and 2. Double knock-out predictions from this pipeline were submitted, placing 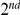 (topology predictions from this pipeline were submitted, placing 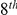). Pipeline 4, Resampling+MCZ, (eq. 22) presents an alternate way to combine predictions made from complementary methods. In pipeline 4 a resampling approach was applied to pipeline 2 and the results were combined with pipeline 1 as described in the methods sections. Topology predictions from pipeline 4 outperformed those of pipeline 3, and double knock-out predictions were on par with those of pipeline 3.

The DREAM4 datasets consisted of both time-series and steady-state data, and participants were challenged to predict: 1) the topology of the network (as a ranked list of regulatory interactions), and 2) the response of the network to combinations of genetic perturbations (double knock-outs). We have participated in both challenges. For challenge 1 we used a relatively simple t-test based method, Median Corrected Z-scores (MCZ, pipeline 1, Figure 1), which tied for 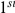 place at predicting the topology of the network. For challenge 2 we used a network inference pipeline (pipeline 3, Figure 1) that combined MCZ with our previously published top-performing method for DREAM3 [31] (tlCLR-Inferelator 1.0, pipeline 2, Figure 1), placing 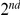 at predicting the response of the network to double knock-outs. Pipeline 3 represents our initial attempt at combining pipeline 1 and pipeline 2 in a mutually reinforcing manner. Although pipeline 3 was complementary to MCZ in that it allowed us to predict the response of the system to double knock-outs, it was not complementary at predicting the topology of the network, placing 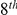 (out of a total of 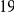 predictions).

After the results for DREAM4 were in, we re-evaluated our methods with the goal of identifying where improvements can be made. We aimed to find an alternate way to combine pipeline 1 and 2 in a mutually reinforcing manner with respect to topology predictions. We show that by combining pipelines 1 and 2 in a resampling approach (pipeline 4, Figure 1), we were able to generate topology predictions that outperformed those of either pipeline. Pipeline 4 also retains the ability to predict the response of the system to double knock-outs. Additionally, we were able to improve upon the ability of pipeline 3 to predict the data (the response of the network to double knock-outs) by reconsidering how we construct the initial conditions. Originally the initial conditions were set to the w.t. expression levels for all genes. We found that alternate initial conditions based on the single gene knock-outs and informed by the MCZ topology predictions were able to achieve an order of magnitude greater data prediction accuracy.

### Problem Set Up

The DREAM4 *in-silico* network reconstruction challenge consists of five synthetic networks of 100 genes used to generate five corresponding datasets. The five networks vary in their topology, chosen to mimic either *Escherichia coli* or *Saccharomyces cerevisiae*, and their dynamical properties, determined by initial conditions and the kinetic parameters chosen for each of the five networks [1]. Stochastic differential equations, followed by the addition of noise proportional to the level of gene expression (as seen in real microarray datasets), were used to generate expression data from each topology.

Denote the expression levels of the genes by 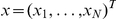. We are given four sets of observations: time-series (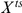), wild-type (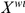), knock-out (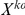), and knockdown (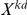). To generate the time-series data a perturbation was introduced into the system for a period of time, and then removed. Measurements were taken at evenly spaced time intervals as the system responded to the perturbation, and as it relaxed. We treated the response of the system to the perturbation and the relaxation of the system once the perturbation was removed as separate time-series experiments. In order to simplify notation and without loss of generality we will assume that 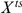 is the result of one such time-series experiment with 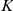 observations, 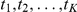, (i.e. 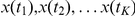 are columns in 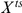). 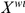 is composed of the first observation in each time series (of which there are ten), and one provided observation of wild-type expression. To generate the knock-out data the transcription rate of each gene was set to zero (in turn), the network was equilibrated, and the steady-state expression for all genes in the system was measured. Likewise, for the knockdown data the transcription rate of each gene was set to half of its wild-type rate, the network equilibrated, and the steady-state expression levels of all of the genes in the system were measured. For the main challenge participants were presented with this data, but not the underlying network topology or kinetic parameters, and asked to submit a ranked list of regulatory interactions sorted by confidence (highest-confidence interactions at the top of the list). The topology predictions were evaluated by area under the precision recall curve (AUPR) [5]. A perfect prediction would have all true regulatory interactions (i.e. true positives) ranked higher than false regulatory interactions (i.e. true negatives), and an AUPR = 1. A random topology prediction for the DREAM4 networks would have an AUPR close to zero.

In addition to this main challenge participants also had the option of taking part in a bonus-round challenge aimed at assessing a method's ability to predict system-wide behavior in response to new genetic perturbations, the double knock-out challenge. For each network participants were also presented with twenty double knock-out perturbations (in which the transcription rate of a pair of genes was set to zero simultaneously), and asked to predict the steady-state expression of all other genes in response to the perturbation. The accuracy of the prediction was evaluated by calculating the mean square error (MSE) between the actual and predicted expression of the 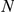 genes. We now describe the three component RN methods which comprise our network inference pipelines: MCZ, tlCLR, and the Inferelator 1.0.

### Core Method 1: Median Corrected Z-scores

The underlying model for the expression data in DREAM4 was generated by stochastic differential equations. Each measurement can be thought of as the observation of only a few cells, as opposed to a population of cells. Accordingly, each measurement of wild-type expression, contained in 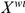, is an estimate of the population wild-type expression derived from only a few samples, making it a relatively noisy observation. Thus, any single observation will not accurately describe the population wild-type expression, and methods that rely on population-wide statistics (such as the t-test) will suffer. A natural way to correct for this is to increase the sample size. By taking the mean (or median) of the expression levels for each gene, 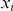, over all wild type observations (11 in total) we can improve our estimate of the population mean. We use the median since it is more robust to outliers than the mean.

We further improved our estimate of the population wild-type expression by taking the median of 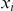 not only with respect to the wild-type observations, 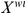, but also with respect to the genetic knock-out data, 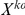. We can do so under the assumption that the networks are sparse (i.e. each gene is regulated by relatively few regulators). Thus, in most single knock-out experiments the level of most genes will be an independent measurement of their wild-type expression. Accordingly, we consider the wild-type expression of gene 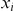 to be the median of its expression in 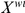 and 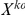, and denote this median-corrected estimate of wild-type expression as 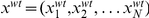.

Previously, we have observed that the genetic knock-out data, 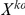, is very informative in regards to the topology of the network [31]. Yip et al. [48] showed that a simple global noise model to filter out non-significant interactions using genetic knock-out data alone was able to produce regulatory interaction ranks of high quality, resulting in the top-performing method for the DREAM3 *in-silico* network challenge. However, for DREAM4 the noise for each gene was a function of the gene's expression (higher noise for higher expression), more accurately simulating the noise found in real microarray measurements. Thus, we used a method that takes into account a more biologically relevant gene-specific noise model to rank regulatory interactions. A natural way of identifying if a gene, 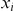, is a target of a TF, 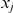, is by comparing the expression level of 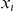 when 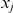 is knocked out to the corrected wild-type expression of 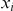, 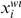. We do so using a median corrected Z-score (MCZ):
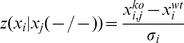
(1)where the notation 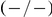 indicates a knock-out experiment (i.e. 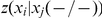 denotes to the MCZ score of target gene 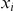 given that 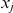 is knocked out), 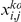 is the expression of gene 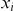 when 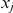 is knocked out, and 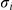 is the standard deviation of gene 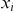 over all wild-type and single gene knock-out observations. We use 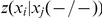 as a measure of confidence for each regulatory interaction 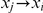, which we store in:
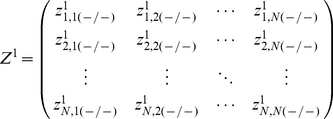
(2)


MCZ performed very well in reconstructing the topology of the network (i.e. ranking regulatory interactions based on confidence), however it cannot be used to learn dynamical models of regulation (and hence cannot be used to make predictions of the system's response to double knock-outs). Additionally, it requires a very complete dataset (knock-out of each gene, in turn) to rank all possible regulatory interactions. Moreover, if a regulator is not highly expressed in the wild type condition then the prediction of its targets using MCZ is not very reliable (in this dataset expression and activity seem to be correlated).

### Core Method 2: Time Lagged Context Likelihood of Relatedness

tlCLR is a MI based method that extends the original CLR algorithm to take advantage of time-series data [29]. tlCLR is more general than MCZ in that it can explicitly use both steady state and time series data to make a prediction of network topology. tlCLR has three main steps: 1) model the temporal changes in expression as an ODE, 2) calculate the MI between every pair of genes, 3) apply a background correction (filtering) step to remove least likely interactions. tlCLR treats all of the steady state data in the same manner. Thus, we combined the three steady state datasets (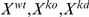) into one 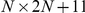 matrix, 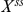 (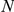 knock-out experiments, 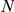 knock-down experiments, and 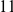 wild type observations).

Mutual information is a metric of dependency between two random variables 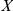 and 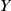, which can be defined as [25]

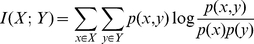
(3)where 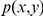 is the joint probability distribution function of 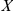 and 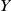, and 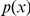 and 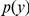 are the marginal probabilities that 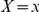 and 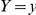, respectively. Note that MI is a symmetric measure. Faith et al. [29] have previously shown that Context Likelihood of Relatedness (CLR), a MI based method, performed well at identifying a large portion of the known *E.coli* regulatory associations as well as predicting novel interactions. However, CLR can only predict undirected regulatory interactions, and must rely on additional data to determine directionality (e.g. by knowing that one gene encodes for a TF and the other for an enzyme, directionality can be resolved). By taking advantage of the temporal information available from time-series observations, we have shown that CLR can be extended (in a method we call tlCLR), allowing us to infer directed regulatory interactions, and improving overall performance [31]. At the core of tlCLR's ability to resolve directionality is its reliance on dynamic-MI instead of static-MI. The computation of static and dynamic -MI is described below.

As previously suggested [26]–[29], MI can be used as a measure of similarity between the expression levels of gene-pairs, 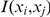, where gene-pairs that show a significantly higher MI scores (compared to other gene-pairs) are more likely to have a regulatory interaction between them. Since 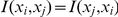 both regulatory edges (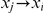 and 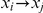) are equally likely. We refer to the MI calculated from 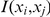 as static-MI, because it does not use the temporal information available from time-series data (treating time-series and steady-state data identically).

### Step 1: Applying an ODE model to the time-series data

We now describe dynamic-MI, which is motivated by our previous work on the Inferelator 1.0 [40], an ODE-based method. We assume that the temporal changes in expression of each gene, 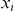, can be approximated by the linear ODE:
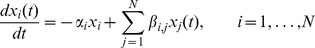
(4)where 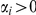 is the first-order degradation rate of 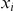 and the 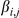
's are a set of dynamical parameters to be estimated. The value of 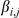 describes the extent and sign of the regulation of target gene 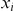 by regulator 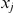. We store the dynamical parameters in an 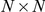 matrix, 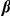. Note that 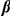 is typically sparse, i.e. most entries are 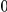 (reflecting the sparsity of transcriptional regulatory networks). Later, we describe how to calculate the values 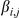 by the Inferelator 1.0. Now we focus on how to use the time-series data in the context of improving the calculation of MI values between a gene 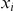 and its potential regulator 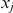. Using a finite difference approximation, we can write (4) for time-series experiments as
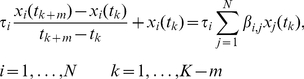
(5)


where 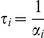 is related to the half-life, 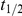, of 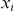 by 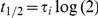, and 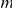 defines the time intervals we consider (e.g. 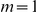 corresponds to time intervals composed of consecutive time-points). We have set 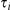 to 50 (for all 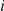), which is the time-interval between measurements, assuming that the sampling frequency was on the time order of most regulatory reactions. For DREAM4 we consider two time intervals: those of length 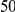 (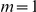) and those of length 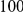 (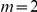). We chose to stop at a time interval of 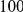 because using longer time intervals did not improve the dynamic predictive performance (as estimated by the Inferelator 1.0 cross validation step which will be described below). Note that the time in the DREAM4 datasets was measured in arbitrary units (i.e. it does not correspond to any of the typical time units: seconds, minutes, hours, etc.).

The purpose of the next two steps is to separate the terms in (5) that involve the putative regulators (the explanatory variables) from the terms in (5) that involve the target (the response variable). We do so first for time-series data and then for steady-state data.

For every gene pair 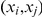, we define a time-series response variable,
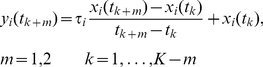
(6)


We pair this response variable with a corresponding explanatory variable, 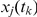. Both variables were derived from the left and right hand sides of (5), respectively.

For steady state experiments, the derivative, 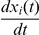, in (4) equals zero, and we can write (4) as
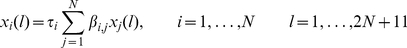
(7)Thus, we define a steady-state response variable,
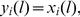
(8)and a corresponding explanatory variable 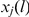, again both derived from (7). Taking the time-series and steady-state response variables together, we get the final response vector:
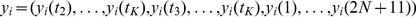
(9)and the final explanatory variables vector:
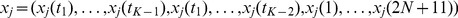
(10)Note that for time-series data the explanatory variables are time-lagged with respect to the response, and that for time intervals much larger than 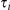 (5) limits to steady state behavior.

To simplify notations, we will define 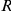 to be the total number of elements in 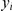 and 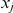, and let 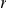 iterate over these entries, 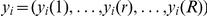 and 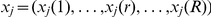, i.e. 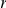 iterates over corresponding response and explanatory variables. We now explain how we use these response (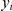) and explanatory (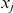) variables to calculate the MI between every pair of genes.

### Step 2: Calculating the dynamic-MI between genes

As a measure of confidence for a directed regulatory interaction between a pair of genes (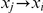) we use, 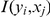, where a pair that shows a high MI score (relative to other pairs) is more likely to represent a true regulatory interaction. Note that 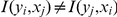, making one regulatory direction more likely than the other. We refer to the MI calculated from 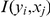 as dynamic-MI, as it takes advantage of the temporal information available from time-series data (distinguishing time-series data from steady-state data).

As described above, we calculate 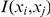 and 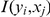 for every pair of genes and store the values in the form of two 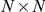 matrices 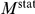 and 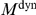, respectively. Note that 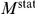 is symmetric while 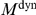 is not. We now briefly describe how tlCLR integrates both static- and dynamic-MI to produce a final confidence score for each regulatory interaction. For a more detailed explanation we refer the reader to [31].

### Step 3: Background correction

For each regulatory interaction 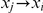 we compute two positive Z-scores (by setting all negative Z-scores to zero): one for the regulation of 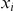 by 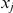 based on dynamic-MI (i.e. based on 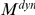),
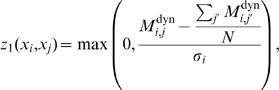
(11)where 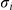 is the standard deviation of the entries in the 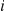
'th row of 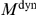. And one for the regulation of 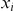 by 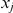 based on both static and dynamic-MI,
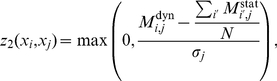
(12)where 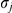 is the standard deviation of the entries in the 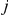
'th column of 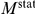. We combine the two scores into a final tlCLR score, 
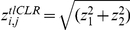
. Note, that some entries in 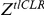 are zero, i.e. 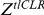 is somewhat sparse. For a more detailed description of tlCLR we refer the reader to [31].

### Core method 3: Inferelator 1.0

We use Inferelator 1.0 to learn a sparse dynamical model of regulation for each gene 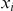. As potential regulators of 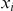 we consider only the 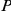 highest confidence (non-zero) regulators. Such a set of 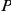 potential regulators can come from any method that ranks regulatory interactions. For example rankings from MCZ, correlation, mutual information, CLR, or tlCLR can all be used to calculate a set of 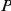 highest confidence regulators of 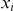. Note that we cannot guarantee that every 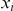 will have 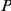 regulators meeting this criteria, thus we denote by 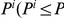) the number of regulators that do. Accordingly, for each gene, 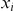, we denote this subset of potential regulators as 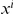. We then learn a sparse dynamical model of regulation for each 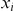 as a function of 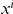
's (using Inferelator 1.0). We assume that the time evolution in the 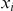
's is governed by
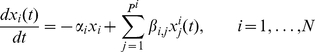
(13)which is exactly (4) with our constraint on the number of regulators. Least Angle Regression (LARS) [39] is used to efficiently implement an 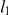 constraint on 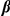, which minimizes the following objective function, amounting to a least-square estimate based on the ODE (13):
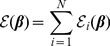
(14)where
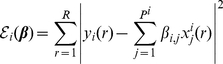
(15)under an 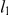-norm penalty on regression coefficients,
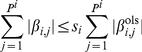
(16)where 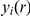 and 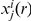 are corresponding elements in the response (9) and design vectors (10), 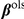 is the over-fit ordinary least-squares estimate (i.e. the minimizer of (15) with no penalty), and 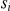 is a number between 0 and 1 referred to as the shrinkage parameter; setting 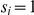 corresponds to ordinary least-square regression. To avoid over fitting, we chose the shrinkage parameter 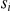 by ten fold cross-validation at one standard deviation away from the minimum error (as described in [32]). Each resultant model (row of 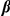) is a parameterization of an ODE describing the temporal evolution of 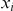. The 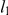 constraint ensures that Inferelator 1.0 results in a sparse matrix, 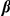, with a small number of entries 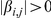. These entries are dynamical parameters that can be used to predict the response of the system to new conditions, such as the removal of genes or future time-points (given initial time points in a time series).

The three methods just described (MCZ, tlCLR, and the Inferelator 1.0) comprise the core network inference methods on which our inference pipelines were built. We now present our four inference pipelines and how they were used to generate topology predictions for the DREAM4 competition. For pipeline 1 (MCZ), a ranked list of regulatory interactions is trivially obtained by using the values in 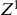 (2). For pipilines 2,3,4 the process of combining multiple methods and generating topology predictions is described below.

### Pipeline 2: combining results from tlCLR and the Inferelator 1.0

The predictions made by pipeline 2 placed 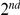 for the DREAM3 competition, but were not submitted for the DREAM4 competition. The reason we did not use pipeline 2 for DREAM4 is that it was outperformed by pipeline 3 on the DREAM3 data. We present pipeline 2 here to simplify our explanation of pipeline 3 (which is a combination of pipelines 1 and 2). When developing pipeline 2 we suspected that predictions made from two different methods (tlCLR, and the Inferelator 1.0) can be complementary. We have previously shown this to be the case [31]. We combined tlCLR and Inferelator 1.0 in two ways: 1) we use the ranked list of regulatory interactions from tlCLR as a feature selection step for the Inferelator 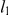 shrinkage approach, and 2) we combined the ranked list generated by tlCLR with the ranked list generated by the Inferelator 1.0.

### Using the tlCLR ranking as a feature selection step for the Inferelator 1.0

The entries of each row, 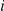, of 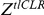 correspond to a ranking of the potential regulators for 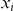. As possible regulators of a gene 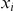 in the Inferelator 1.0 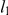 model selection step (15) we used the 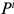 most likely regulators from row 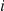 of 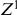. In this way we used the ranking of regulatory interactions predicted by tlCLR as a feature selection step for the Inferelator 1.0. We then combined the ranked list of regulatory interactions generated by each of the two methods. 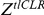 can be used to rank regulatory interactions based tlCLR. We note that prior to combining the results of tlCLR with those from the Inferelator 1.0 we employed a simple filtration step where we removed all regulatory interactions that had MCZ scores in the lower 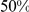 of all MCZ scores. We now describe how a ranked list of regulatory interactions was calculated by the Inferelator 1.0.

### Calculating a ranked list of regulatory interactions by the Inferelator 1.0

The dynamical parameters stored in 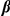 (the result of minimizing (15) subject to (16)) describe the regulation of each target gene as a function of its regulators (TF's) in the system, with 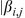 corresponding to the strength of the regulation, and the sign of 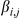 indicating repression or activation. For the DREAM3 *in-silico* challenge we ranked regulatory interactions using 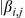 as the measure of confidence for a regulatory interaction (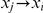) [31]. However, this ranking does not take into account the explanatory power of each predictor 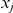 in the ODE model for a target 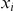 (e.g. 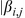 may be large even though the model for the regulation of 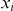 is not a good one). Here, we propose a confidence measure that incorporates the explanatory power of predictors (i.e. the quality of the model for 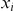).

Denote the predicted expression of 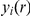 as 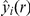, calculated as 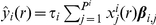. The error in this approximation of 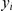 was measured as sum-of-squares, 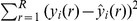, where 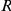 is the number of elements in the response vector, 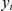. We estimated the predictive error of our model for 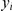 using mean error obtained from ten fold cross-validation. In order to place all model errors on the same scale, we normalized the absolute sum-of-squares error to derive a measure of relative error, 
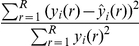
. Given this relative error, we defined the explanatory power of the model for 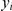 to be given by 1 minus relative error:
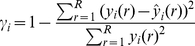
(17)where 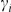 represents the merit of the model for 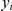 (i.e. how good of an estimate is 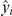). We can now calculate the contribution of each predictor 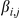 to the explanatory power of the model for 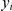, (i.e. the explanatory power of each regulatory interaction) as a weighted average
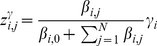
(18)where 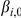 is the bias term for the regulatory model of 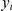. Note that here we use the fact that all the observations of the regulators 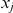
's, are on the same scale, as they were normalized to have zero mean and standard deviation of 1 before model selection by Inferlator 1.0 (a common step in a regression framework). We stored these values in the form of an 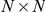 matrix, 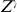, which can be used regulatory interactions based on Inferelator 1.0 alone. However, based on our previous results that the predictions made by different methods may be complementary, we combined the predictions made by tlCLR (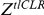) with those made by Inferelator 1.0 (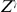).

### Combining topology predictions made by tlCLR with those made by the Inferelator 1.0

The main challenge in combining these confidence scores is that they are not guaranteed to be on the same scale. Thus, we developed a single rank based heuristic (described previously in [31]) to combine two separate sets of confidence scores. Our approach is best explained by an example: Let 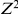 denote the resultant matrix from combining the confidence scores contained in 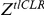 and 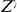, i.e. the results our tlCLR-Inferelator pipeline (pipeline 2, Figure 1). We first replace the value of the highest-ranking entry in 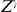 with the value of the highest-ranking entry from 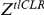. We then replace the value of the second highest-ranking entry from 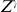 with the value of the second highest-ranking entry from 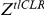. We continue in such a way until all non-zero entries in 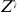 have been replaced by equally ranked entries in 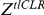. This produces two ranked lists of regulatory interactions that are on the same scale. Once this assignment is done we can combine the two matrices as follows:
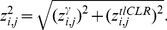
(19)Note that here 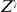 refers to the matrix after the assignment of values from 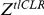. 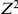 constitutes the results of applying pipeline 2. Pipeline 3 is very similar to pipeline 2. In order to assess how complementary tlCLR-Inferelator and MCZ were we combined the confidence scores stored in 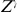 with those in 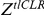 (replacing scores of equal ranks from 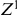 into 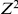, as above):
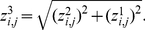
(20)The confidence scores contained in 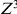 were generated by a combination of our three methods, and we will refer to this integrated method as tlCLR-Inferelator+MCZ (pipeline 3, Fig 1). 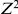 and 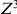 can be used as ranked lists to rank regulator interactions for pipelines 2 and 3, respectively.

### Combining genetic and dynamic information in a resampling approach

We generated an ensemble of networks as follows. Denote by 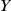 the 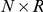 response matrix, with each row set to 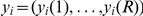. Similarly, denote by 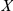 the 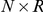 design matrix, with each row set to 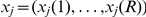. Let 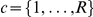 be the vector of column indices for both 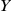 and 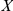. We sample with replacement 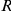 times from 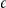, storing the selected indices in 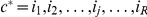, 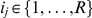. We now consider the permuted data matrices, 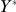 and 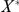, comprised of the 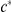 columns of 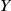 and 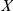 respectively. We generate 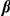, 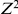, and 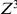, as described before, with the only difference being that we use the response and explanatory vectors from 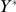 and 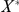, respectively, instead of 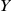 and 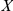. We repeat this procedure 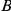 times, with 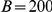 for the DREAM4 networks, each time generating 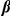, 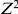, and 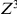. We store this ensemble of regulatory network predictions in:
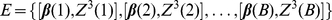
(21)where 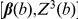 corresponds to the dynamical parameters and rankings based on tlCLR-Inferelator+MCZ (pipeline 3, Figure 1) at resample 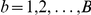. Note that throughout this resampling procedure the ranks generated by MCZ remain constant. We used this ensemble of network predictions by selecting, for each regulatory interaction 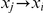 (corresponding to entries 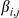 and 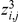), the median dynamical parameters in 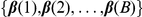 and the median tlCLR-Inferelator+MCZ rank in 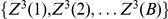. We store these median values in 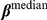 and 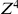, respectively. These matrices have entries:
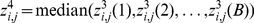
(22)

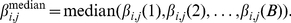
(23)We used 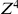 to rank the regulatory interactions, and we refer to this resampling approach as Resampling+MCZ (pipeline 4, Figure 1). 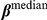 is a set of dynamical parameters which can be used to predict the response of the system to new perturbations (such as the simultaneous knock-out of two regulators).

### Bonus round: Generating the double knock-out predictions

The challenge of predicting the response of the system to double knock-outs (double-KO) can be phrased as: given a simultaneous knock-out of two genes (i.e. 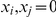 for some 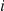 and 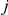), predict the steady-state expression of all other genes. In order to predict steady-state expression levels for each gene we used the steady-state limit of the core Inferelator 1.0 model [32] (7), which we rewrite here (in matrix notation) for the case of predicting the steady state data:
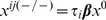
(24)where 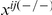 is the level of all genes for the double-KO of genes 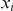 and 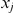, and 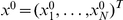 is some vector of initial conditions (satisfying 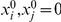). Note, that for DREAM4 we made a simplification, setting 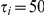 for all 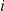, i.e. we assume that all mRNA have the same half-life. The only unknown left to determine (in order to make a prediction) is the vector of initial conditions, 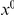. The rest of this section deals with computing a good initial condition vector.

A simple way to pick this vector would be to set 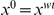, with the exception that 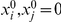. The results that we submitted for the DREAM4 bonus-round challenge were calculated using this initial condition. Note, however, that the system's response to the double-KO of genes 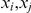 individually was already given to us in the single-gene knock-out dataset, 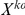. Upon revisiting our initial results, after submission of the predictions, we reasoned that using the single gene knock-out (single-KO) information to predict double-KO expression would most likely yield better results, as it reflects a system state that is closer to the state we are trying to predict. Indeed, using the single-KO data to determine initial conditions markedly improved the accuracy of our double knock-out predictions.

One simple approach to construct initial conditions from the single gene knock-outs of 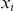 and 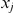 is to simply take their mean. However, we chose to use a more informed approach by taking advantage of our previous knowledge regarding likely regulatory interactions (i.e. the confidence scores from MCZ (stored in 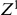). We do so by computing the following weighted average:
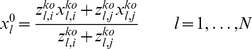
(25)where 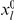 is our estimate for the initial expression level of gene 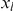, 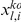 and 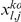 are the observed levels of 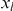 when genes 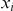 and 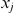 were knocked out, respectively, and 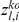 or 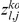 are the confidence scores (calculated by MCZ) for each regulatory interaction 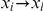 and 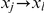, respectively. In this manner we computed an initial condition vector, 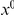, for every double-KO we were asked to predict. We then used these initial conditions to calculate a prediction of the expression of all genes in the presence of a double-KO of 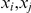 via (24). We denote this prediction as 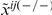.

Note that some models had more predictive merit than others, as measured by the explanatory power of each model (17). Thus we weighted the prediction of double knock-outs by the predictive merit of each model. We computed the final double-KO predictions as follows:
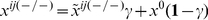
(26)where 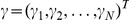. Note that in (26) the final prediction 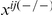 is weighted by our estimate of the predictive performance of the models, 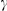 calculated in (17), and constrained, using the initial conditions, by our estimate of the model errors 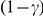.

## Results

### Performance of tested methods: ranking putative regulatory interactions

The main challenge in the DREAM4 100 gene *in-silico* regulatory network competition was to predict the topology of five networks. Predictions were made in the form of a list of regulatory interactions ranked in decreasing order by confidence. We evaluated the performance of four pipelines for learning regulatory networks, namely: MCZ (pipeline 1, eq. 2), tlCLR-Inferelator (pipeline 2, eq. 19), tlCLR-Inferelator+MCZ (pipeline 3, eq. 20), and Resampling+MCZ (pipeline 4, eq. 22). We developed these pipelines with a focus on combining results from multiple methods in a mutually reinforcing manner. In all four cases we evaluated the quality of the rankings of all possible regulatory interactions using the area under precision recall curve (AUPR), as this was the basis for the evaluation of performance in DREAM3 and DREAM4.

**Figure 2:**
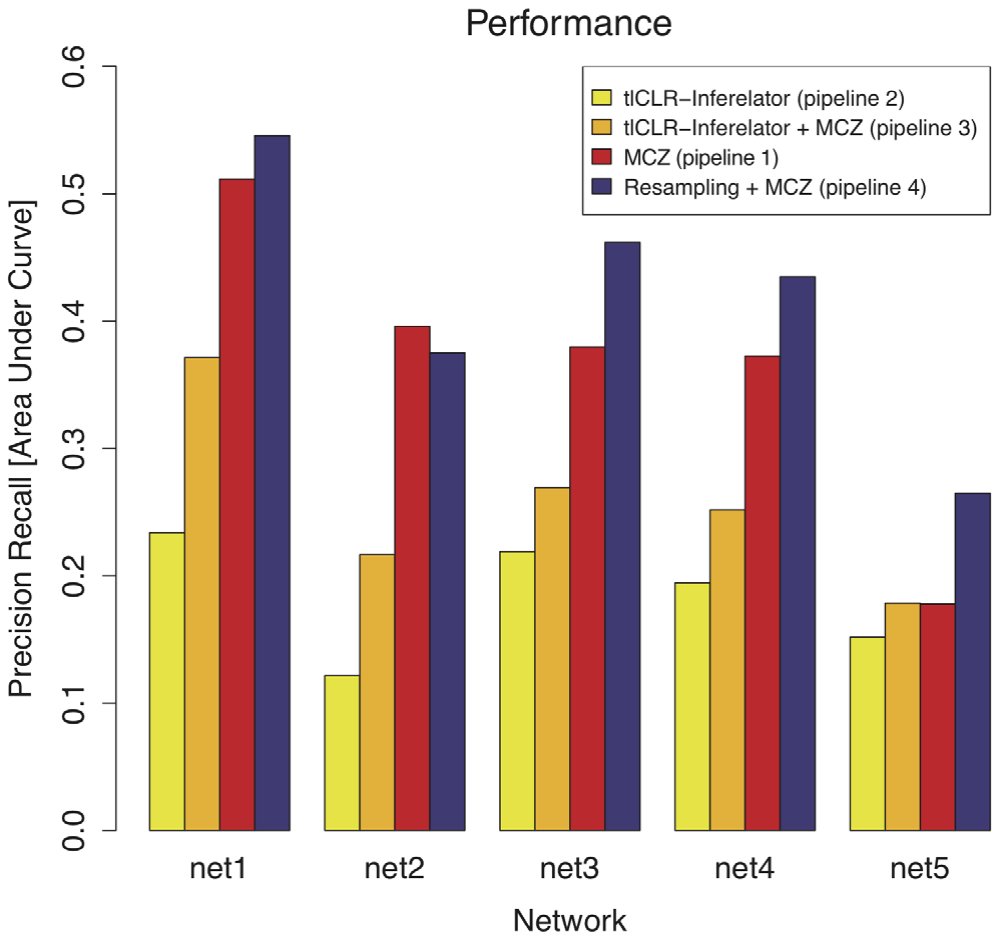
Area under precision recall curve for each ranking scheme. For each pipeline we evaluated the performance in predicting topology using area under the precision recall curve (AUPR). We see that pipeline 4 generally outperforms all other methods, followed by MCZ, pipeline 3, and pipeline 2.

We submitted the results of MCZ as our ranked list of regulatory interactions for the DREAM4 challenge. This method tied for first place (out of 19 teams). In Figure 2 we see that pipeline 2 exhibits lower performance for most of the networks. In pipeline 3 we combined the predictions made by pipeline 1 with those made by pipeline 2. As expected for methods that are not complementary, the performance of pipeline 3 is better than that of pipeline 2 but worse than that of pipeline 1. However, by using a resampling approach, pipeline 4 (eq. 22), to generate an ensemble of likely networks we see a marked improvement over the performance of any other method (Figure 2, purple bars). This improvement is most evident in networks 3–5, which appear to be more difficult to predict for all of the methods we tested.

### Performance of methods based on genetic knock-out data decreases with decreasing expression of the regulators

**Figure 3:**
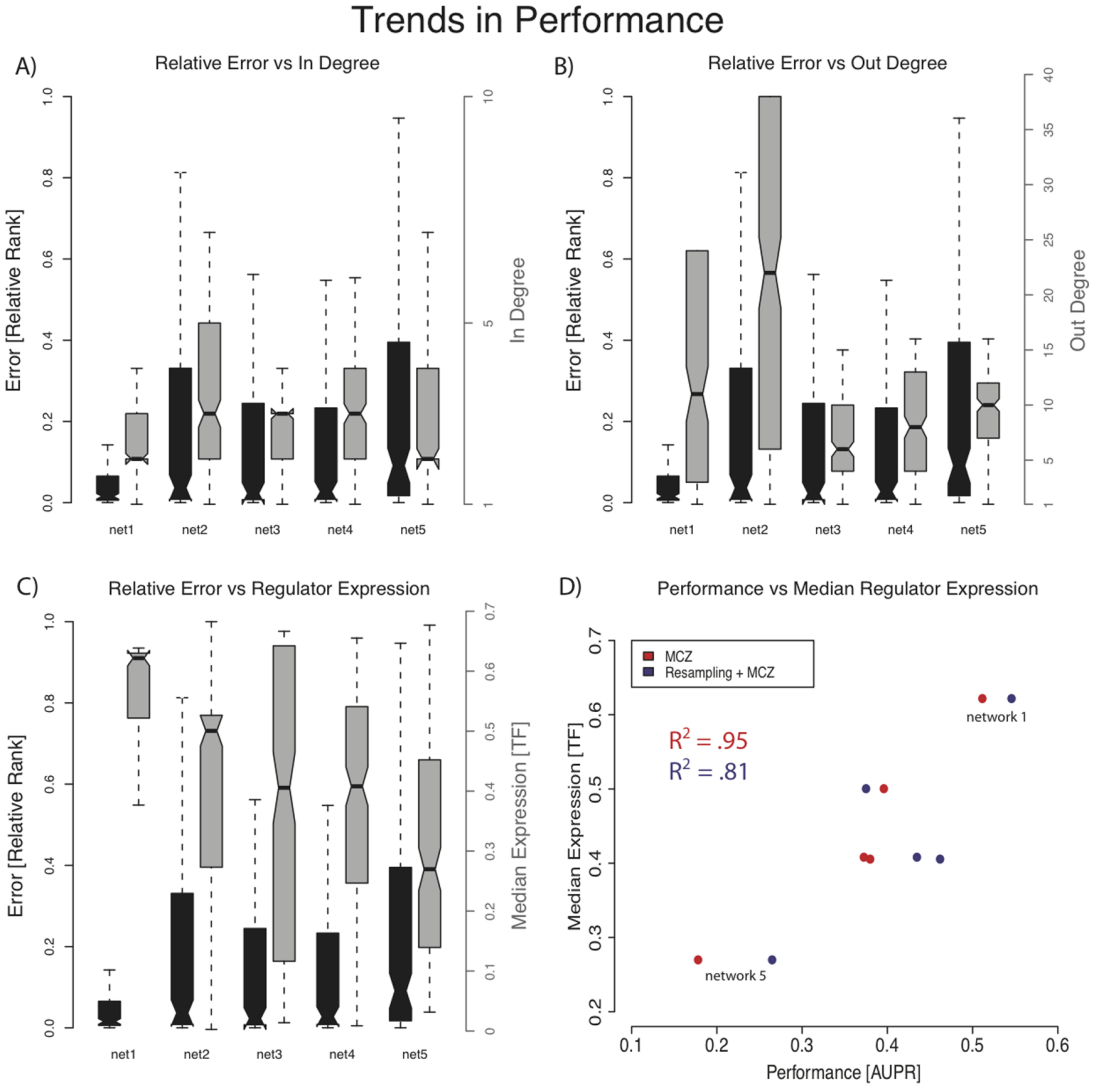
Trends in performance over the five networks. For panels A,B,C we consider only the performance of MCZ, and use relative rank as an estimate of error. We compute relative rank in the following way. Denote by 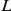 the total number of possible regulatory interactions, and by 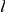 the rank that was given to each regulatory interaction, 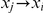. The relative rank of 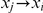 is defined to be 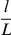. Error distributions of the predictions for the five networks are shown as black boxplots in panels A,B,C. Distributions of in-degree of the regulators, out-degree of the regulators, and median expression of the regulators are shown as gray boxplots in panels A,B,C, respectively. **A**) There is no apparent relationship between relative rank (Error) and the in-degree of the regulators. **B**) There is no apparent relationship between relative rank (Error) and the out-degree of the regulators. **C**) Relative rank (Error) in network prediction increases as the median expression of the regulators decreases. **D**) we show the relationship between median expression of the regulators and the performance in ranking regulatory interactions, in terms of AUPR, across all five networks. For MCZ a correlation of (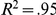) exists between the TFs median expression and AUPR (shown in red), while for Resampling+MCZ there is a smaller correlation of (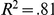) between the TFs median expression and AUPR (shown in purple).

For the DREAM3 *in-silico* challenge all methods, including several similar to the ones we test herein, were found to perform significantly worse for networks with very high in-degree (targets regulated by many TFs) and to be relatively insensitive, performance-wise, to the out-degree of TFs [31], [49]. We did not find this trend in the current challenge (Figure 3A,B). However, we did find that performance varies considerably across the five 100 gene networks for all tested methods; performance was best for the first network and dramatically worse for the fifth network (Figure 2). We investigated possible reasons for this, finding that performance is correlated with the median expression of the regulators. Given a regulatory interaction, 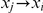, our chance of correctly predicting that regulatory interaction (based on MCZ) tends to be higher if the median expression of 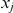 over all conditions in the knock-out data-set, 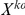, is high. Conversely, the smaller the median expression of 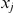, the worse our performance. Figure 3C shows that our predictions for the regulatory interactions in network 1 have relatively low error (black box plot), and the corresponding median expression of the regulators in this network is relatively high (gray box plot). For network 5 we see a relatively high error, and the corresponding median expression of the regulators in this network is the lowest of the five networks. In Figure 3D we see a high correlation, 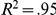, between the median expression of the regulators and the performance of MCZ in terms of AUPR. By combining ranks from MCZ with our resampled network inference pipeline, pipeline 4, we significantly improve performance on networks 3–5 (Figure 2), and lower the correlation between performance and median TF expression over all five networks to 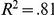 (Figure 3D).

### Regulatory interaction rankings derived from genetic knock-out data and rankings derived from resampling pipeline 2 are complementary

In the above section we focused on differences between the performance of each method for each of the five networks. In this section we focus on the performance of each method in a gene-by-gene manner, in an effort to better understand how to best utilize heterogeneous data collections. Specifically, we investigated the performance of each network inference pipeline as a function of the median expression of the regulators in the network. We bin regulators based on their median expression, and compare the error made in predicting their respective targets.

**Figure 4:**
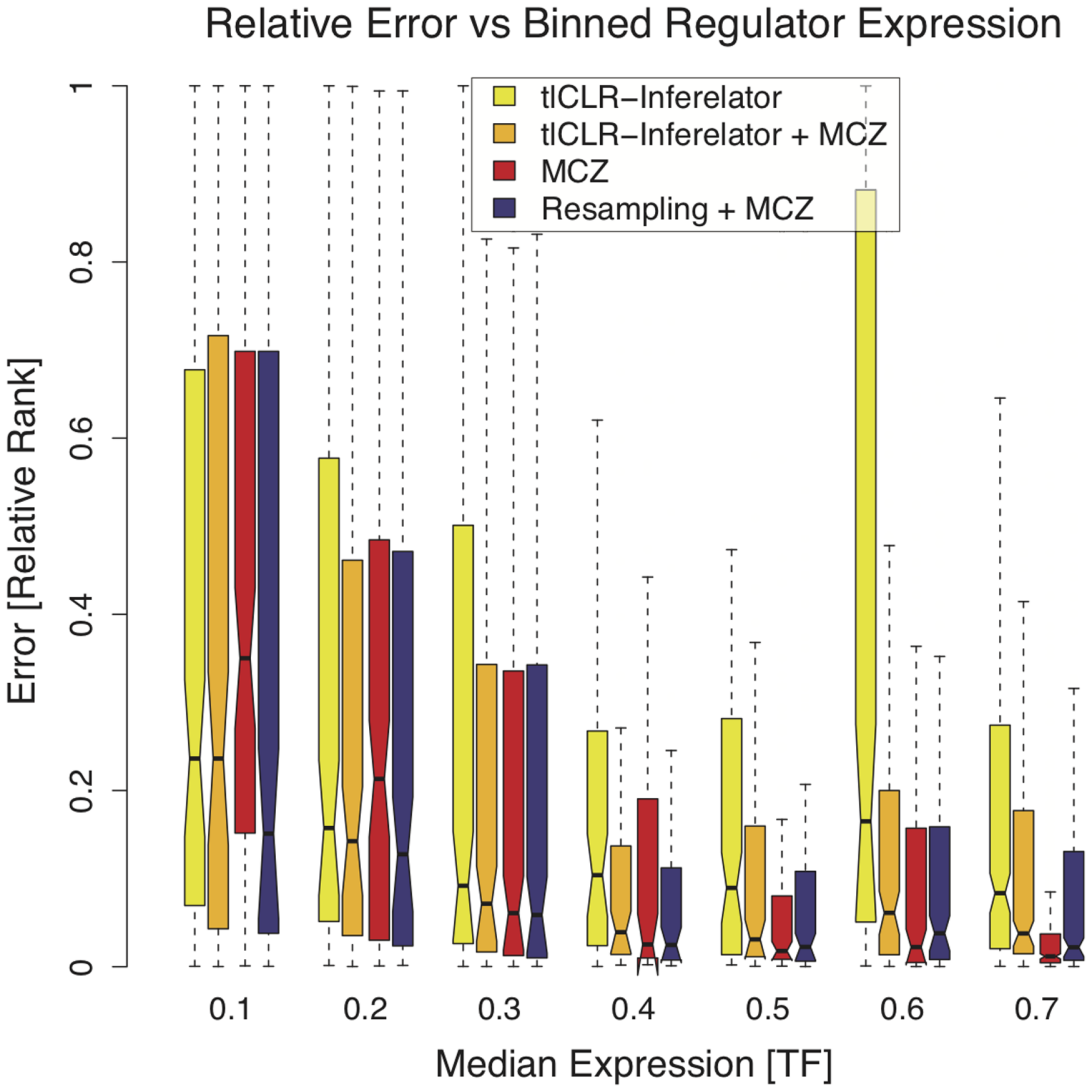
Error as a function of binned median expression for all regulatory interactions. We further investigate the relationship between the median expression of the regulators and each pipeline's performance in predicting topology. We use relative rank as an estimate of error (as in Figure 3). We bin the regulators for all five networks based on their median expression (each of the seven bins has a roughly equal number of regulators). We show the distribution of relative ranks (Error) for each pipeline in each bin of regulator expression. We see that all of the pipelines that incorporate the predictions of tlCLR-Inferelator (pipelines 2,3, and 4) outperform MCZ for regulators with low median expression (bins 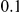, 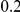).

In Figure 4 we see that the performance of MCZ is better for regulators with a higher median expression (shown in red). This trend is more apparent in this gene-by-gene view than in our network-centric analysis. Looking at each bin, shown from low to high median expression, we see that predictions made by pipelines that incorporate rankings made by tlCLR-Inferelator perform better than the predictions made by MCZ for regulators whose median expression is low (bins 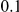, 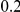). The error distributions of the predictions made by pipeline 4 (purple bars) are lower than those of MCZ (red bars) for regulators with a median expression upto 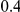, and on par with the predictions made by MCZ for regulators with a median expression of up to 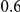. The predictions made by pipeline 4 are better than those made by pipelines 2 and 3 for all bins.

### Predicting response of the system to double knock-out

For each 100-gene network we were asked to predict the cell's steady-state mRNA levels given that a pair of genes is knocked out. There are twenty such pairs of genes (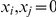) for each network. We make these predictions using the parameterization, 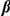, of the system obtained from pipeline 3 (tlCLR-Inferelator+MCZ). We also make these predictions using the parameters obtained by taking the median weight from the ensemble, 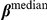 (eq. 23), generated by pipeline 4 (Resampling+MCZ). The measure of performance for the DREAM4 double knock-out predictions was mean squared error (MSE). As a baseline, we compare the error of our prediction to the error we would make if we used the initial conditions as the prediction.

**Figure 5:**
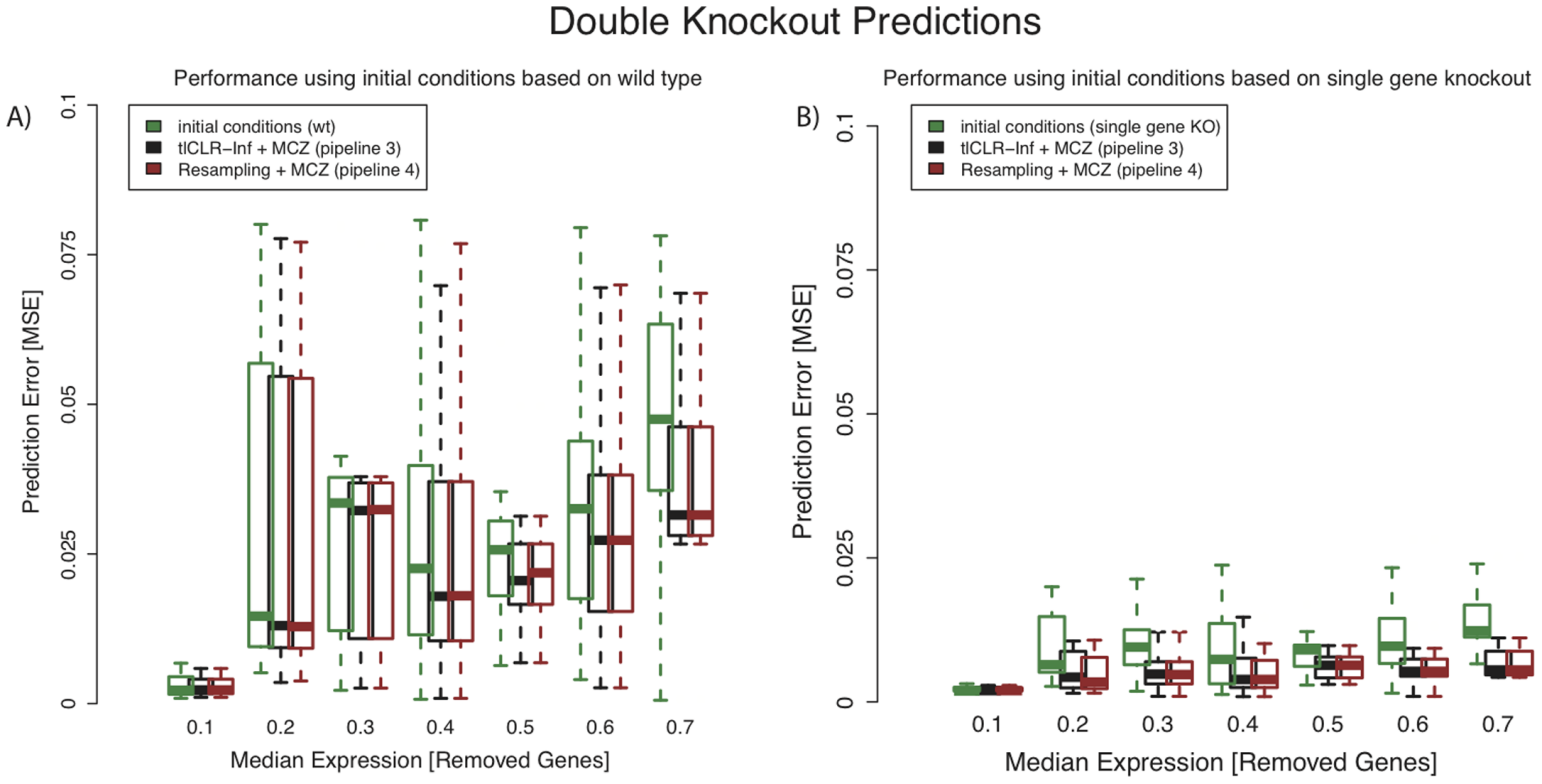
Performance on double knock-out prediction. We assess the accuracy of predicting the system's response to the simultaneous removal (knock-out) of two genes 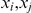. In total, there were one-hundred pairs of genes that were knocked out. We bin these pairs of genes based on the average of their respective median expression in the single-gene knock-out data. We made two predictions, which differ only in the choice of initial conditions. We compare the error (as evaluated by the mean squared error) of our prediction to the error made by using the respective initial condition as a prediction. **A**) We use the wild-type expression, 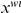, as the set of initial conditions (green boxplots). We see that our predictions (black and red boxplots) are more accurate than if we used the initial conditions as a prediction (this is more apparent for TFs with a larger median expression). **B**) We use a combination of the single-gene knock-outs to compute our initial conditions (eq. 25). We do this because the single-gene knock-out data represents a system state that is closer to the state we are trying to predict than wild-type (as can be observed by comparing the green boxplots in panel A to those in panel B). We show the error distributions using parameters calculated by either pipeline 3 (tlCLR-Inferelator+MCZ) or pipeline 4 (Resampling+MCZ), gray and red boxplots, respectively, are smaller than the error distributions if we used the initial conditions as a prediction. Regardless of the choice of initial conditions, the error distributions using parameters calculated by pipeline 4 (red boxplots) are similar to the error distribution obtained by pipeline 3.

In Figure 5 we bin regulators based on their median expression and show the corresponding error distributions for our predictions. We compare our error to the error made if we used the initial conditions as a prediction of the response of the system. In Figure 5A we use the wild type expression, 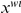, as the set of initial conditions. We see that predictions made using either pipeline 3 (gray boxplots) or pipeline 4 (red boxplots) outperform the initial conditions (green boxplots). In Figure 5B we construct our initial conditions from the given single gene knock-out values and our MCZ confidence scores (eq. 25). We see that our predictions (black and red boxplots) outperform the initial conditions (green boxplots). Furthermore, by comparing the green boxplots in Figure 5B to those in Figure 5A, we see that predictions based on initial conditions derived from the single knock-out data have much lower error than predictions based on initial conditions derived from the wild type. Regardless of which initial conditions are chosen, predictions using parameters derived from pipeline 4 show almost identical performance as those made by using the parameterization derived from pipeline 3.

## Discussion

We participated in the DREAM4 100-gene *in-silico* network inference competition. The method that we submitted, and that was the co-best performer on the 100-gene *in-silico* challenge, was the rankings derived from the median corrected Z-scores of the genetic knock-out data, MCZ. The power of the genetic knock-out data, as also shown by Yip et al. in DREAM3 [48], is an important point to consider for experimental design. However, it does have limitations for which we compensated by integrating other data-types, particularly time-series data. We observed that as the median expression of the regulators in a network decreases, error in predicting regulatory interactions using MCZ increases (Figure 3). A plausible explanation for why a low median expression of regulators leads to poor performance is that if a regulator that is more likely to be active (i.e. a regulator whose wild-type expression is high) is removed, then the corresponding effect on its targets will be relatively large. Conversely, if a regulator that is less likely to be active (i.e. its wild-type expression is low) is removed, then the effect on its targets will be marginal. Perhaps, the targets of such regulators will be most apparent in over-expression experiments. If over-expression experiments are not available, the poor performance in predicting the targets of these regulators can be mitigated by combining the predictions made by MCZ with predictions made by a method that takes advantage of time-series data.

We used pipeline 3 (tlCLR-Inferelator + MCZ), which takes advantage of the time-series data, to predict topology and dynamical parameters for each network in a way that was more robust to the median expression of the regulators than methods that use solely genetic knock-out data. We submitted topology predictions and bonus-round (double knock-out) predictions generated by pipeline 3. The topology predictions ranked 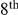 out of 19 teams. Pipeline 3 is an improvement upon pipeline 2 in terms of AUPR (Figure 2). Pipeline 2 ranked 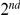 out of 22 on the DREAM3 100-gene network inference challenge [31]. However, pipeline 3 ranked 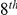 on the DREAM4 challenge. This discordance between a worse performance relative to other teams, but improved ability to recapitulate network topology is probably due to a more concentrated use of the knock-out data by participants of DREAM4.

Upon receiving the gold-standard networks we analyzed our ability to rank regulatory interactions using the different pipelines. Dissecting our performance, in a gene-by-gene manner, we saw the that there are instances when predictions made by pipeline 3 are more accurate than those made by MCZ. Given the performance of each of the methods, as evaluated by AUPR (Figure 2), this is a surprising and promising result, implying that methods that use only genetic knock-out data and those that take advantage of time-series data produce complementary topology predictions. Further demonstrating this point, we showed that applying a resampling approach to pipeline 3 and combining the results with MCZ, by aggregating the ranks derived from each method, produces a final prediction that is better than the predictions generated by either method alone. The improvements from resampling (pipeline 4) are most evident on networks 3–5 (Figure 2), which have the lowest median expression of the regulators (Figure 3A), and are hence hardest to predict using the genetic knock-out data alone. We note that alternate ways of combining predictions from multiple methods may further improve upon our results. We also note that in pipeline 4 the predictions of MCZ remain constant for each network in the ensemble. This implies that although a single network generated by pipeline 3 may perform poorly, our resampling approach generates sufficient alternate topologies such that picking a network based on the ensemble-median produces a much more accurate topology prediction. This resampling approach also infers an ensemble of dynamical parameters, retaining the ability to predict the response of the network to new conditions.

We submitted predictions of system-wide expression in the presence of double knock-outs for the DREAM4 bonus-round challenge. The predictions we submitted were based on the initial conditions derived from wild-type expression levels (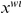). The quality of our double knock-out predictions was very sensitive to the initial conditions (Figure 5). We found that using the single gene knock-out data together with MCZ confidence scores as the basis of our initial conditions dramatically improves our predictive performance (compared to using initial conditions based on the wild-type). This is due to the fact that the single-gene knock-outs present a closer network state to the state we are trying to predict (network response to double knock-outs) than does the wild-type. When using initial conditions based on the single gene knock-out data we saw that our improvement over these initial conditions was larger than when using initial conditions based on wild type (Figure 5). This is an interesting observation since one might expect that it would be harder to improve upon initial conditions that are already close to the true answer. We accurately predicted the response of the network to double knock-outs using dynamical parameters calculated by Pipeline 3 (whose topology prediction was poor relative to those of other methods). Thus, we show that our ability to predict data can tolerate a remarkable amount of error in the predicted topology and still make accurate predictions of the system's response to new perturbations. This is perhaps not surprising, as the Inferelator 1.0 [32] was designed to minimize data prediction error. We have also shown that a parameterization picked from the median of an ensemble of networks (generated by resampling pipeline 3) retains, but does not significantly improve, our ability to predict data in the double knock-out challenge (in spite of the fact that this method produces more accurate topology predictions). Perhaps an alternative way of picking parameters from the ensemble of networks can improve upon the ability to predict new data.

We have shown the complementarity between predictions made using genetic knock-out data and those made using time series data. We have shown that using solely genetic knock-out data can result in accurate topology predictions, which can be further improved upon by correctly incorporating predictions made using time-series data. To this end, we have developed a relatively simple method for combining the predictions made from genetic knock-out and time-series datasets, showing an improved ability to infer network topology while maintaining the ability to predict the response of the system to new conditions. We suggest that investigating alternate means of combining genetic and dynamic experimental designs (leveraging the complementarity between these two data-types), as well as methods that incorporate direct binding data, will continue to be fruitful avenues of future investigation.

**Table 1:** Salient characteristics of the three core methods.

	*MCZ*	*tlCLR*	*Inf*
**input data**			
optimal for comprehensive knock-out data	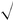		
optimal for ts data		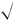	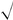
**output**			
topology (directed network)	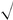	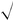	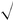
kinetic parameters (can predict system's response)			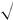
**statistical approach**			
t-statistic	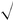		
mutual information		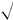	
regression			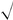

Here we present the characteristics of the three core network inference methods, combinations of which constituted network inference pipelines. Median corrected z-score (MCZ) uses the t-statistic on solely the steady state genetic knock-out data, and can predict the topology of the network. Time-lagged Context Liklihood of Relatedness (tlCLR) is a mutual information based method that uses both time-series and steady state data to predict the topology of the network. The Inferelator 1.0 (Inf) is an ordinary differential equation (ODE) based method that uses both time-series and steady-state and can predict not only the topology of the network but also the kinetic parameters of regulation, allowing for the prediction of the response of the system to new conditions.
